# Supplementary material for: Feasibility of the Xemio app for breast cancer survivors in a clinical setting: Adherence, acceptance, and side effect monitoring (CTCAE vs. QoL)
Source: PLoS One. 2026 May 13;21(5):e0342702. doi: 10.1371/journal.pone.0342702 (PMC13170889; doi:10.1371/journal.pone.0342702)
Supplement: S1 File — (DOCX) [file pone.0342702.s001.docx]

**Xemio App Evaluation Ad-hoc questionnaire**

**mACMA-ASCAPE study- AD-HOC questionnaire**

In this questionnaire we ask you to rate your experience using the XEMIO application.

GENERAL QUESTIONNAIRE

1. It has been easy to use the application

| Totally disagree |  |  |  | Totally agree |
| --- | --- | --- | --- | --- |
| 1 | 2 | 3 | 4 | 5 |

2. It has been able to orient itself within the application

| Totally disagree |  |  |  | Totally agree |
| --- | --- | --- | --- | --- |
| 1 | 2 | 3 | 4 | 5 |

3. The application has allowed you to track symptoms

| Totally disagree |  |  |  | Totally agree |
| --- | --- | --- | --- | --- |
| 1 | 2 | 3 | 4 | 5 |

4.It has been easy to assess the intensity of their symptoms

| Totally disagree |  |  |  | Totally agree |
| --- | --- | --- | --- | --- |
| 1 | 2 | 3 | 4 | 5 |

5. It has been easy to add treatments

| Totally disagree |  |  |  | Totally agree |
| --- | --- | --- | --- | --- |
| 1 | 2 | 3 | 4 | 5 |

6. It has been easy to find events

| Totally disagree |  |  |  | Totally agree |
| --- | --- | --- | --- | --- |
| 1 | 2 | 3 | 4 | 5 |

7. You have found events of interest to you

| Totally disagree |  |  |  | Totally agree |
| --- | --- | --- | --- | --- |
| 1 | 2 | 3 | 4 | 5 |

8. You have found the information you have consulted in the app useful

| Totally disagree |  |  |  | Totally agree |
| --- | --- | --- | --- | --- |
| 1 | 2 | 3 | 4 | 5 |

9.Hygienic-dietary advice has helped to alleviate her symptoms.

| Totally disagree |  |  |  | Totally agree |
| --- | --- | --- | --- | --- |
| 1 | 2 | 3 | 4 | 5 |

1. OPEN-ENDED QUESTIONS

10. What did you like most about Xemio?

11. What did you like least about Xemio?

1. FEATURES

12. Which features have you used the most? 5 is a lot, 0 is nothing

[  ] Consult information

[  ] Symptom registry

[  ] Drug registry

[  ] Physical activity

[  ] Calendar of events

1. EVALUATION

13.Would you recommend the application?

Yes/ No

14. Why?
