# Supplementary material for: Feasibility of the Xemio app for breast cancer survivors in a clinical setting: Adherence, acceptance, and side effect monitoring (CTCAE vs. QoL)
Source: PLoS One. 2026 May 13;21(5):e0342702. doi: 10.1371/journal.pone.0342702 (PMC13170889; doi:10.1371/journal.pone.0342702)
Supplement: S2 File — (DOCX) [file pone.0342702.s002.docx]

**APP’s side effects measures by oncological categories CTCAE**

Oncological categories: All intensity values are grouped into 4 oncological categories, which are:

1. Mild -> the patient is given hygienic-dietary advice.
2. Moderate -> the advice includes the recommendation to consult their specialist doctor.
3. Severe -> the patient should go to the medical center if they experience a symptom with severe intensity.
4. Vital Urgency -> the patient must seek immediate medical attention due to the life-threatening nature of the symptom.

| Side Effect’s list | Intensity values | |
| --- | --- | --- |
|  | min | max |
| Abdominal pain | 1 | 3 |
| Acne breakout | 1 | 4 |
| Anorexia | 1 | 4 |
| Anxiety | 1 | 4 |
| Bone pain | 1 | 3 |
| Chills | 1 | 3 |
| Conjunctivitis | 1 | 3 |
| Constipation | 1 | 4 |
| Decreased libido | 1 | 2 |
| Depression | 1 | 4 |
| Diarrhea | 1 | 4 |
| Difficult digestion | 1 | 3 |
| Difficulty swallowing | 1 | 4 |
| Dry eye | 1 | 3 |
| Dry mouth | 1 | 3 |
| Dry skin | 1 | 3 |
| Fatigue | 1 | 3 |
| Fever | 1 | 3 |
| Flu-like symptoms | 1 | 3 |
| Gastroesophageal reflux | 1 | 3 |
| Gastrointestinal pain | 1 | 3 |
| Generalized muscle weakness | 1 | 3 |
| Gingival pain | 1 | 3 |
| Hair loss | 1 | 2 |
| Headache | 1 | 3 |
| Hemorrhoidal bleeding |  |  |
| Hot flashes | 1 | 3 |
| Hypertension | 1 | 4 |
| Insomnia | 1 | 3 |
| Intermittent chest pain | 1 | 3 |
| Joint pain | 1 | 3 |
| Lymphedema | 1 | 3 |
| Memory loss | 1 | 3 |
| Menopause | 1 | 3 |
| Metallic taste | 1 | 2 |
| Mouth sores (mucositis) | 1 | 4 |
| Muscle cramp | 1 | 3 |
| Muscle pain | 1 | 3 |
| Nail involvement | 1 | 1 |
| Nausea | 1 | 3 |
| Oral pain | 1 | 3 |
| Painful intercourse | 1 | 3 |
| Palmar-plantar syndrome | 1 | 3 |
| Photosensitivity | 1 | 4 |
| Stomach pain | 1 | 3 |
| Syncope | 1 | 1 |
| Tingling or numbness | 1 | 4 |
| Toothache | 1 | 3 |
| Vaginal dryness | 1 | 3 |
| Vaginal pain | 1 | 3 |
| Vomiting | 1 | 4 |
| Weight gain | 1 | 3 |
| Weight loss | 1 | 3 |
