# Supplementary material for: Feasibility of the Xemio app for breast cancer survivors in a clinical setting: Adherence, acceptance, and side effect monitoring (CTCAE vs. QoL)
Source: PLoS One. 2026 May 13;21(5):e0342702. doi: 10.1371/journal.pone.0342702 (PMC13170889; doi:10.1371/journal.pone.0342702)
Supplement: Attachment — Submitted filename: Response to Reviewers.pdf [file pone.0342702.s005.pdf]

Response to Reviewers

Manuscript ID: PONE-D-25-09322
Title: *Feasibility of the Xemio App for Breast Cancer Survivors in a Clinical Setting: Adherence, Acceptance, and Side Effect Monitoring (CTCAE vs. QoL)*

Dear Academic Editor and Reviewers,

We sincerely thank you for your thoughtful comments and suggestions, which have substantially improved the clarity and rigor of our manuscript. Below we provide a point-by-point response. All changes in the revised manuscript are marked with track changes (red deletions, blue insertions).

Editorial Requirements

When submitting your revision, we need you to address these additional requirements.

Comment: ***1. PLOS ONE style and file naming***

Response: *Done. The manuscript was reformatted using the PLOS ONE templates, and file names were corrected accordingly.*

Comment: 2. **Role of funders**
Response: The Funding Statement now reads:

> "This research work was carried out as part of the EU-funded Research and Innovation Action ASCAPE (Project ID: 875351). The improvement of the Xemio-Research platform was supported by a La Caixa Foundation Grant (LCF/PR/AR19/51450002). **The funders had no role in study design, data collection and analysis, decision to publish, or preparation of the manuscript.**"

Comment: 3. **Funding text in Acknowledgments**

Response: Funding information was removed from the Acknowledgments. The Funding Statement remains as above.

> "This research work was carried out as part of the EU-funded Research and Innovation Action ASCAPE (Project ID: 875351). The improvement of the Xemio-Research platform was supported by a La Caixa Foundation Grant (LCF/PR/AR19/51450002). **The funders had no role in study design, data collection and analysis, decision to publish, or preparation of the manuscript.**"

Please include your amended statements within your cover letter; we will change the online submission form on your behalf.

Response: We updated the statement to read:

Comment: 4. **Competing Interests**
Response: The statement now reads:

> "I have read the journal's policy and the authors of this manuscript have the following competing interests: Maria-Angeles Fuentes-Expósito and Imma Grau-Corral are affiliated with the iSYS Foundation, the organization responsible for developing and promoting the Xemio-Research App. **This does not alter our adherence to PLOS ONE policies on sharing data and materials.**"

Comment: 5. **Data sharing restrictions (policy compliance)**
Response (a–b):

- **(a) Ethical/legal restrictions.** The dataset includes potentially identifying clinical and sociodemographic information from breast cancer survivors. Public release would contravene the EU GDPR (2016/679), the Spanish Organic Law 3/2018, and the protocol approved by the Ethics Committee for Drug Research of Hospital Clínic de Barcelona (CEIm-HCB; **HCB/2020/0971**).

- **(b) Access mechanism.** Qualified researchers may request controlled access via **CEIm-HCB (CEIm@clinic.cat)**. Requests will be evaluated for GDPR/protocol compliance and confidentiality safeguards; if approved, a **minimally necessary, de-identified** dataset will be shared under a **Data Use Agreement**.

Comment: 6. **Online form—Data Availability language**

Response: We removed "reasonable request to the author" and replaced it with the **committee-mediated** access route described above. See final Data Availability Statement below.

Comment: **7. Copyright/permissions for figures**

Response: We confirm that all figures (Fig 1, Fig 2, and the Striking Image) are original screenshots and graphical materials of the Xemio-Research app, developed by **Fundación iSYS**, which is the copyright holder. We have obtained explicit permission from Fundación iSYS to publish these figures under the **CC BY 4.0 license** and have updated the figure captions accordingly:

- **Fig 1. Xemio App Interface.** The figure shows a user holding a smartphone displaying the Xemio app, developed by Fundación iSYS. Reprinted under a CC BY license, with

permission from Fundación iSYS, original copyright 2024.

- **Fig 2. Patient-reported side effect screens in the Xemio app.** Examples of the user interface for patient-reported symptom tracking in the Xemio mHealth app. (a) Reporting painful intercourse, where users can adjust the intensity level using a color-coded slider. (b) Reporting dry skin, where users select affected body areas and can indicate additional symptoms like rash or pus. (c) Reporting vomiting, where users choose intensity levels based on predefined episodic frequency categories. These features allow real-time symptom tracking, facilitating personalized patient management. Reprinted under a CC BY license, with permission from Fundación iSYS, original copyright 2020.

- **Striking Image.** Visual representation of the Xemio app. Reprinted under a CC BY license, with permission from Fundación iSYS, original copyright 2024.

We will also upload the signed **PLOS Content Permission Form** as an "Other" file, confirming that Fundación iSYS authorizes publication of these figures under the CC BY 4.0 license.

Comment: 8. **Reviewer-suggested citations**

Response: We reviewed suggested works and added citations where directly relevant to mHealth survivorship adherence, digital engagement barriers, and CTCAE–PROMs complementarity, avoiding unnecessary citation density.

Comment: 9. **Reference list integrity**

Response: Checked for completeness, formatting, and retraction status. Minor formatting corrected; no retracted works cited.

Reviewers' comments:

Comment: 3. Have the authors made all data underlying the findings in their manuscript fully available?

***Response**: We thank both reviewers for flagging this. We have revised our **Data Availability Statement** to fully comply with PLOS policy:*

- ***What cannot be public:** The individual-level dataset contains potentially identifying clinical and sociodemographic information from breast cancer survivors collected under protocol **HCB/2020/0971** at **Hospital Clínic de Barcelona**. Public release would contravene the **EU GDPR** and the approved informed consent reviewed by the **Ethics Committee for Drug Research of Hospital Clínic de Barcelona (CEIm-HCB)**.*

- ***Formal access route: Qualified researchers** may request controlled access via **CEIm-HCB (CEIm@clinic.cat)**. Requests will be evaluated for GDPR compliance, protocol/consent alignment, and confidentiality safeguards; if approved, a **minimally***

***necessary, de-identified dataset** will be shared under a **Data Use Agreement**.*

Reviewer #1:

Comment: Manuscript is well-structured and suitable; minor edits suggested.

***Response:** Thank you. We conducted a global language/consistency review, harmonized decimal punctuation (international English), improved parallel structure in lists, and rechecked figure numbering and cross-references.*

Reviewer #2:

Comment: The article is a prospective feasibility study evaluating the Xemio-Research mobile health application for breast cancer survivors, with a focus on adherence, usability, and patient-reported outcomes. The authors have conducted a one-year longitudinal study within the ASCAPE project, reporting descriptive analysis of user engagement, usability scores, and symptom tracking.
The manuscript in overall is technically sound and presents a well-structured descriptive statistics study to evaluate the app's usability, adherence, and patient-reported outcomes. However, several revisions are recommended:

- (a) more explicitly acknowledge the limitations of the small, convenience-based sample size and the absence of formal inferential analyses;

Response: Addressed in **Study design and participation** and **Limitations**: we explicitly state the **convenience-based, single-center sample** and that the analysis is primarily **descriptive**.

- (b) clarify that the findings are exploratory and feasibility-focused rather than confirmatory;

Response: Added in **Data Analysis**, **Discussion**, and **Limitations** that findings are **exploratory and feasibility-focused**, not confirmatory.

- (c) improve the Data Availability Statement to comply with the journal's policy; and

Response: We have revised the Data Availability Statement to fully comply with PLOS ONE policy. The updated statement clarifies that individual-level data cannot be shared publicly due to ethical and legal restrictions (EU GDPR, Spanish Organic Law 3/2018, and the protocol approved by the Ethics Committee for Drug Research of Hospital Clínic de Barcelona, CEIm-HCB; protocol HCB/2020/0971). Instead, qualified researchers may request controlled access by contacting CEIm-HCB at **CEIm@clinic.cat**. Requests will be reviewed for compliance with GDPR, protocol, and confidentiality safeguards, and data will be shared under a Data Use Agreement if approved.

- (d) address minor language, formatting, and readability issues.

***Response:** Implemented all requested edits (see details below).*

Comment: The statistical analysis is appropriate for a feasibility study, with the focus on

descriptive statistics (means, standard deviations, and frequencies). The analysis aligns with the study's stated objectives. The use of validated instruments including the System Usability Scale (lines 213–216) strengthens the study approach. However, the analysis could be enhanced in several aspects.

- First, no inferential testing was conducted, such as in areas where simple comparisons (e.g., CTCAE vs. QoL reporting, lines 252–296) could be analysed with chi-square, correlation analyses, or Cohen's kappa. Confidence intervals are not reported, which limits interpretation of uncertainty. In addition, handling of missing data is not discussed, despite clear reduction of participants over time (lines 178–184, 196–208). I recommend that the authors emphasize that this is an exploratory, feasibility-focused study but not a hypothesis testing. Adding basic inferential or agreement analyses will be helpful to strengthen the validity of the findings.

Response: We acknowledge the reviewer's suggestion. As this was an exploratory feasibility study with a small, single-arm sample, no inferential testing or hypothesis-driven analyses were planned. The Data Analysis section now explicitly states that the study focused on descriptive, feasibility-oriented analyses and complete-case reporting without imputation, consistent with feasibility study standards.

- Apart from that, following the PLOS Data Policy, the current Data Availability Statement does not meet the required standard. The manuscript indicates that data cannot be shared publicly due to privacy regulations, and that access may be granted upon reasonable request to the author. While the sensitivity of patient data is acknowledged, PLOS requires that authors either deposit de-identified data in a public repository (setting up a controlled access if necessary) or provide a clear explanation of restrictions accompanied with a formal mechanism for data access (for example through an institutional or ethics committee). In this sense, author discretion via "reasonable request" is insufficient. I recommend that the authors revise the Data Availability Statement to ensure compliance with the journal policy.

Response: We have fully revised the Data Availability Statement to comply with the PLOS Data Policy. The updated statement now provides:

- A **formal ethical and legal justification** for not releasing individual-level data publicly (due to GDPR, Spanish Organic Law 3/2018, and the study protocol approved by the Ethics Committee for Drug Research of Hospital Clínic de Barcelona, CEIm-HCB; HCB/2020/0971).

- A **formal access mechanism** through CEIm-HCB (CEIm@clinic.cat), which evaluates data requests for compliance with GDPR, protocol, and confidentiality safeguards.

- A commitment to share a **minimally necessary, de-identified dataset** under a Data Use Agreement if approval is granted.

- While the discussion provides a summary of the main findings, I find the interpretation somewhat superficial. For example, the decline in adherence (lines 304–316) has been acknowledged and compared to other digital health interventions, but the analysis

ended before exploring underlying reasons. Potential factors such as digital literacy, socioeconomic background, treatment-related fatigue, or psychological barriers are not addressed, despite relevant based on the demographic characteristics and the disease.

Response: We expanded the **Discussion** to consider potential factors related to the observed decline in adherence—**digital literacy**, **socioeconomic context**, **treatment-related fatigue**, and **psychological burden**—and connected these to prior mHealth survivorship literature. We also discuss **practical implications** for clinic workflows (e.g., integrating brief staff prompts, tailoring notification frequency, onboarding support) and **sustainability** (e.g., aligning usage windows with clinical milestones, offering opt-in **gamification** features, and **just-in-time adaptive** reminders). Citations have been added to substantiate these points.

- Similarly, gamification and real-time monitoring are suggested as possible solutions. these ideas are presented briefly without linking to existing evidence or theory. I recommend that the authors expand the discussion to deeply analyze the factors associated with the decline of adherence, connect findings to prior literature on mHealth in survivorship care, and discuss practical implications for clinical adoption and sustainability.

Response: We grounded these recommendations in relevant literature on digital engagement and patient-reported outcomes in oncology and survivorship, and clarified that our suggestions are **implementation-focused hypotheses** for future testing.

Comment: Overall, the manuscript is clearly written. I suggest a few minor edits to further improve clarity and readability.

- In lines 110–116, the description of the sample size could be clarified by explicitly noting that this was a convenience sample.

***Response**: Now explicitly states **"convenience sample"** and single-center recruitment.*

- In lines 144–146, shifting the phrasing from future to past tense ("the analysis focused on...") would ensure consistency with the Results section.

Response: Revised to past tense ("**the analysis focused on...**").

- At line 197, the sentence could be streamlined to "Adherence was analyzed through frequency of app usage" for conciseness.

Response: We agree that conciseness improves readability. However, adherence in our study was not based only on generic "app usage," but specifically on two metrics: (1) frequency of side-effect reports and (2) number of days with recorded step data. To balance clarity and precision, we revised the sentence to:

> **"Adherence was assessed using two metrics: the frequency of side effect reports and the number of days with recorded step data."**

This streamlined phrasing reflects the actual operational definition used while keeping the text concise.

We found the duplicate heading occurred because one section described *how adherence was measured* (Methods) and the other reported *what we found* (Results). To avoid redundancy and improve clarity, we:

1. **Methods → Adherence assessment (renamed):** kept only the operational definition of adherence (two metrics: frequency of side-effect reports and number of days with recorded step data).

2. **Results → Adherence results (renamed):** kept only the outcomes (quarterly use and engagement figures).

These edits remove the duplicated title, align content with section purpose (methods vs. findings), and follow PLOS ONE structure.

The list of negative feedback items in lines 233–239 would read more smoothly if expressed in a parallel structure (e.g., “compatibility issues, difficulties in symptom tracking, information overload, technical problems, perceived impersonality”).

Response: We thank the reviewer for the suggestion. The list of negative feedback items has been revised into a parallel structure for conciseness: *“smartphone compatibility issues, difficulties in symptom tracking, information overload, technical problems, perceived impersonality.”*

To retain nuance from patient feedback, we added a clarifying sentence noting that some participants perceived impersonality as a constant reminder of their illness

- In lines 299, the phrase “feasibility and acceptability” is repeated unnecessarily and could be reworded for variety.

Response: We thank the reviewer for the helpful observation. To avoid redundancy and improve readability, we replaced *“The Xemio feasibility study assessed the feasibility and acceptability ...”* with the more concise phrasing *“This study, conducted from November 2020 to November 2022 in the context of the ASCAPE project, evaluated the patient acceptance and usability of the Xemio-Research App ...”*.

- Finally, the conclusion in lines 359–367 might be softened to indicate that the app “has the potential to support survivorship outcomes,” which more accurately reflects the exploratory, descriptive nature of the data.

Response: We thank the reviewer for the suggestion. To better reflect the exploratory and descriptive nature of this feasibility study, the conclusion has been softened. It now states that the Xemio-Research App *“has the potential to support breast cancer survivorship care”* rather than *“underscores the feasibility and acceptability ...”*.

[NOTE: If reviewer comments were submitted as an attachment file, they will be attached to this email and accessible via the submission site. Please log into your account, locate the manuscript record, and check for the action link "View Attachments". If this link does not appear, there are no attachment files.]

While revising your submission, please upload your figure files to the Preflight Analysis and Conversion Engine (PACE) digital diagnostic tool, https://pacev2.apexcovantage.com/. PACE

helps ensure that figures meet PLOS requirements. To use PACE, you must first register as a user. Registration is free. Then, login and navigate to the UPLOAD tab, where you will find detailed instructions on how to use the tool. If you encounter any issues or have any questions when using PACE, please email PLOS at figures@plos.org. Please note that Supporting Information files do not need this step.

- **Suggestions for Grammar, Style, and Clarity**

1. *Comment:* Abstract

- Original: "Participants tracked symptoms, reported side effects, and engaged with the app."
  Suggestion: Add a clearer subject or timeframe: "During the study period, participants tracked symptoms, reported side effects, and engaged with the app."
- Original: "The app provided higher granularity in symptom tracking..."
  Suggestion: Use more precise language: "The app enabled more detailed symptom tracking…"

***Response**: Added timeframe subject: "**During the study period,** participants tracked symptoms...". Replaced "higher granularity" with "**enabled more detailed symptom tracking**."*

2. *Comment:* Introduction

- Sentence: "The long-term and late effects of breast cancer survivors and its treatment entail..."
  Correction: "The long-term and late effects experienced by breast cancer survivors and the treatments they undergo entail..."
- Clarify this sentence: "Notably, the International Consortium for Health Outcomes Measurement (ICHOM)..."
  Suggestion: Include a clearer link to the app's content: "Notably, ICHOM's standardized PROMs for breast cancer informed the Xemio app's development and symptom domains."

Response: Clarified phrasing on long-term effects ("**experienced by breast cancer survivors...**"). Linked ICHOM to app content ("**ICHOM's standardized PROMs... informed the Xemio app's development...**").

3. *Comment:* Methods

- Sentence: "ASCAPE collects health-related quality of life scores for EORTC QLQ-C30..."
  Suggestion: Revise for smoother syntax: "The ASCAPE project collects HRQoL scores using validated instruments, including…"

- Sentence: “The recruitment period started on December 1, 2020...”
  Suggestion: Add clarity about duration: “Patient recruitment occurred from December 1, 2020 to December 22, 2021.”

Response: Revised ASCAPE sentence (“**collects HRQoL scores using validated instruments, including...**”). Clarified recruitment window (“**from December 1, 2020 to December 22, 2021**”).

4. *Comment:* Results

- Sentence: “The SUS score, derived from the adjusted responses...”
  Suggestion: Add brief context: “The SUS score, calculated from responses to the 10-item usability questionnaire, was...”
- Minor: Throughout the results, ensure consistent use of decimal punctuation (e.g., 82.78 not 82,78 if targeting international English conventions).

Response: SUS phrasing revised (“**calculated from responses to the 10-item usability questionnaire**”). Harmonized decimal punctuation (e.g., **82.78**).

5. *Comment:* Discussion

- Sentence: “This trend is common in digital health interventions...”
  Suggestion: If available, add a citation or more specific explanation of comparable apps.
- Sentence: “The CTCAE complements these tools by enabling precise tracking of detailed symptoms...”
  Suggestion: “...by allowing precise, real-time tracking of specific symptoms not captured in traditional instruments.”

Response: Added supporting citation for the statement that declining engagement is common in digital interventions line 324, and refined the CTCAE sentence to “**allowing precise, real-time tracking of specific symptoms not captured in traditional instruments.**”

6. *Comment:* Conclusion

- Sentence: “The insights gained from participant feedback and symptoms highlight...”
  Suggestion: Consider clarity: “Participant feedback and symptom reports underscored the importance of continuous refinement...”

Response: Revised to “**Participant feedback and symptom reports underscored the importance of continuous refinement...**”

- **Minor Formatting Issues**

- *Comment:* Ensure consistent use of spaces after punctuation throughout (some sections lack spacing after full stops or commas).
- *Comment:* Figures referred to as "Fig 2" are inconsistent with numbering in the text. Double-check all figure references.
- *Comment:* Line spacing and header hierarchy (e.g., subheadings) could be better formatted for visual readability.

Response: Standardized spacing after punctuation and verified all **figure numbering** and cross-references. Improved **line spacing and header hierarchy** for readability within PLOS layout constraints.

- **Additional Recommendations**

1- Enhancing Clarity:

- *Comment:* Consider expanding the explanation of how the app's CTCAE-based tracking improves clinical integration compared to traditional PROMs. A comparative strengths/limitations table could help.

Response: Added a **comparative strengths/limitations table** in the **Discussion** (Table `table_comparative`) and expanded text explaining how **CTCAE-based, real-time, patient-prioritized tracking** complements **standardized PROMs** for clinical decision-making.

2- Reviewer Appeal:

- *Comment:* You may consider briefly discussing data privacy handling mechanisms, as this is a critical point in digital health acceptance.

Response: We appreciate this valuable suggestion. In the revised manuscript, we have added a brief discussion on data privacy handling mechanisms as an essential factor for the acceptance of digital health interventions. Specifically, we now highlight that:

- All data collected in the study were pseudonymized and stored securely within the Hospital Clínic infrastructure (ONTO-CR), with access restricted to authorized clinical staff.

- Data processing complied with the **EU GDPR (2016/679)** and the **Spanish Organic Law 3/2018**, as well as the study protocol approved by the **Ethics Committee for Drug Research of Hospital Clínic de Barcelona (CEIm-HCB; HCB/2020/0971)**.

- Transfers outside the local infrastructure involved only anonymized, encrypted data, never identifiable information.

- These measures were explained to participants during the informed consent process, supporting both compliance and trust, which are central to digital health acceptance.

3- Limitations Section:

- *Comment:* While COVID-19's impact is mentioned, clarify whether any follow-up strategies (e.g., remote support, telephone guidance) mitigated these disruptions.

Response: In Limitations, we now state that we used remote support and periodic telephone reminders to mitigate disruptions, while acknowledging these could not fully offset pandemic-related challenges.

We hope these revisions fully address the reviewers' and editor's comments and that the improved manuscript meets PLOS ONE standards for publication. We appreciate the opportunity to revise and resubmit our work.

Sincerely,

María Ángeles Fuentes-Expósito, Imma Grau-Corral, and co-authors
