# Supplementary material for: Feasibility of the Xemio app for breast cancer survivors in a clinical setting: Adherence, acceptance, and side effect monitoring (CTCAE vs. QoL)
Source: PLoS One. 2026 May 13;21(5):e0342702. doi: 10.1371/journal.pone.0342702 (PMC13170889; doi:10.1371/journal.pone.0342702)
Supplement: Attachment — Submitted filename: Reviewer comments.docx [file pone.0342702.s003.docx]

The manuscript titled “***Feasibility of the Xemio App for Breast Cancer Survivors in a Clinical Setting: Adherence, Acceptance, and Side Effect Monitoring (CTCAE vs. QoL)”*** effectively presents a well-structured and insightful evaluation of a digital health tool in breast cancer survivorship. The manuscript is generally well-prepared and suitable for publication in its current form. However, a few minor revisions could enhance the clarity and impact of the findings. Therefore, I recommend acceptance for publication following the submission of these minor corrections.

- **Strengths**
- Scientifically relevant and timely topic with real-world application.
- Clear objectives and well-defined methodology.
- Rich use of validated instruments (SUS, CTCAE, EORTC QLQ).
- Tables and figures provide detailed support for findings.
- **Suggestions for Grammar, Style, and Clarity**

1. Abstract

- Original: "Participants tracked symptoms, reported side effects, and engaged with the app."
  Suggestion: Add a clearer subject or timeframe: “During the study period, participants tracked symptoms, reported side effects, and engaged with the app.”
- Original: "The app provided higher granularity in symptom tracking..."
  Suggestion: Use more precise language: “The app enabled more detailed symptom tracking…”

2. Introduction

- Sentence: "The long-term and late effects of breast cancer survivors and its treatment entail..."
  Correction: “The long-term and late effects experienced by breast cancer survivors and the treatments they undergo entail...”
- Clarify this sentence: "Notably, the International Consortium for Health Outcomes Measurement (ICHOM)..."
  Suggestion: Include a clearer link to the app’s content: “Notably, ICHOM’s standardized PROMs for breast cancer informed the Xemio app’s development and symptom domains.”

3. Methods

- Sentence: “ASCAPE collects health-related quality of life scores for EORTC QLQ-C30...”
  Suggestion: Revise for smoother syntax: “The ASCAPE project collects HRQoL scores using validated instruments, including…”
- Sentence: “The recruitment period started on December 1, 2020...”
  Suggestion: Add clarity about duration: “Patient recruitment occurred from December 1, 2020 to December 22, 2021.”

4. Results

- Sentence: “The SUS score, derived from the adjusted responses...”
  Suggestion: Add brief context: “The SUS score, calculated from responses to the 10-item usability questionnaire, was...”
- Minor: Throughout the results, ensure consistent use of decimal punctuation (e.g., 82.78 not 82,78 if targeting international English conventions).

5. Discussion

- Sentence: “This trend is common in digital health interventions...”
  Suggestion: If available, add a citation or more specific explanation of comparable apps.
- Sentence: “The CTCAE complements these tools by enabling precise tracking of detailed symptoms...”
  Suggestion: “...by allowing precise, real-time tracking of specific symptoms not captured in traditional instruments.”

6. Conclusion

- Sentence: “The insights gained from participant feedback and symptoms highlight...”
  Suggestion: Consider clarity: “Participant feedback and symptom reports underscored the importance of continuous refinement...”
- **Minor Formatting Issues**
- Ensure consistent use of spaces after punctuation throughout (some sections lack spacing after full stops or commas).
- Figures referred to as “Fig 2” are inconsistent with numbering in the text. Double-check all figure references.
- Line spacing and header hierarchy (e.g., subheadings) could be better formatted for visual readability.
- **Additional Recommendations**
  1- Enhancing Clarity:
- Consider expanding the explanation of how the app’s CTCAE-based tracking improves clinical integration compared to traditional PROMs. A comparative strengths/limitations table could help.
  2- Reviewer Appeal:
- You may consider briefly discussing data privacy handling mechanisms, as this is a critical point in digital health acceptance.
  3- Limitations Section:
- While COVID-19's impact is mentioned, clarify whether any follow-up strategies (e.g., remote support, telephone guidance) mitigated these disruptions.
